# Nummular keratopathy in a patient with Hyper-IgD Syndrome

**DOI:** 10.1186/1546-0096-7-14

**Published:** 2009-08-05

**Authors:** Courtney L Kraus, Susan M Culican

**Affiliations:** 1 Washington University School of Medicine, 660 South Euclid Ave, Saint Louis, Missouri 63110, USA; 2 Department of Ophthalmology and Visual Sciences, Washington University School of Medicine, Saint Louis, Missouri 63110, USA

## Abstract

**Purpose:**

To report a case of recurrent nummular keratitis in a pediatric patient with Hyperimmunoglobulinemia D syndrome.

**Methods:**

A retrospective chart review.

**Results:**

A 14-year-old boy with Hyperimmunoglobulinemia D syndrome (HIDS) presented with photophobia and ocular irritation concomitant with disease exacerbation. He was found on exam to have significant nummular keratitis, which responded to a short course of topical steroids. Despite acute response to local immunosuppression, the patient had several recurrent attacks and eventually developed a large corneal scar and decreased vision. After initiation of infliximab therapy his ocular sequelae improved dramatically and his vision returned to 20/20.

**Conclusion:**

One possible form of end-organ damage associated with HIDS is vision threatening nummular keratopathy.

## Background

Hyperimmunoglobulinemia D syndrome (HIDS) results from mutations in the mevalonate kinase (MVK) gene resulting in deficiency of MVK, an important enzyme for cholesterol biosynthesis. Patients with this rare condition characteristically experience recurrent episodes of fever and inflammation.[1] Starting in the first year of life, patients have periodic fever episodes with accompanying lymphadenopathy, gastrointestinal complaints, aphthous ulcers, arthralgias, and skin reactions. These attacks typically last 4–6 days and recur every 4–6 weeks. We report herein a case of HIDS with photophobia secondary to significant nummular keratitis that resulted in decreased vision and corneal scarring.

A mutation in the MVK gene can affect enzymatic activity of MVK and results in one of two distinct syndromes, HIDS or mevalonic aciduria. In patients with HIDS, the activity of MVK is reduced to 5–15% of normal. The resulting clinical picture may include any or all of the aforementioned symptoms. Laboratory analysis reveals a vigorous acute-phase response during attacks; including elevated erythrocyte sedimentation rate, C-reactive protein and leukocytosis. Most patients have increased serum immunoglobulin (Ig) D and A levels. Mevalonic aciduria, an autosomal recessive disorder with complete absence of MVK activity, usually presents with a more severe clinical picture. It is characterized by periodic attacks of fever and inflammation, along with developmental delay, ataxia, and dysmorphic features. Ocular symptoms consist of retinal dystrophy, moderate optic atrophy and nuclear cataracts.[2] However, specific ocular surface inflammation has not been described previously in either condition associated with MVK mutations, and there are no reports of ocular sequelae of HIDS.

MVK is essential for isoprenoid metabolism, which has end products such as cholesterol, ubiquinone and dolichol, as well as a role in isoprenylation of proteins.[3] How a deficiency of mevalonate kinase leads to inflammation is incompletely understood. The clinical picture of HIDS is believed to reflect enhanced proinflammatory cytokine production by mononuclear cells.[4] A defect in apoptosis of lymphocytes has also been detected.[5] The proinflammatory state, with resultant overproduction of tumor necrosis factor alpha (TNF-α) and interleukin 1-beta (IL1β), is believed to induce the disease's characteristic attacks.[3]

## Report of case

A 14-year-old white boy presented to the St. Louis Children's Hospital Emergency Department with primary complaints of fever, arthritis, and flu-like symptoms of one week's duration. He had been diagnosed 3 years prior with HIDS after a long history of similar presentations. Laboratory examination at this visit revealed IgD levels to be 265 IU/ml (upper limit of normal <100 IU/mL). Genetic testing subsequently established he was a compound heterozygote for two common mutations in the MVK gene, V377I in exon 10 and I268T in exon 8 (GeneDx, Inc, Gaithersburg, MD). At the time of our initial encounter, he was being followed by a rheumatologist. His flares were managed with ibuprofen as needed.

His ocular history was significant for multiple episodes of "eye inflammation" that occurred in conjunction with his febrile attacks. In the past, he had been told he likely had conjunctivitis secondary to viral illness; however he had not been evaluated by an ophthalmologist. During this presentation we were consulted for his new complaint of photophobia and ocular irritation. At our initial exam, visual acuity was 20/50 in the right eye and 20/40 in the left. Both eyes had conjunctival injection on external examination. Slit-lamp exam revealed multiple punctate anterior corneal stromal opacities with active peripheral corneal stromal keratopathy consistent with sterile inflammatory nummular keratitis. We prescribed topical prednisolone acetate and cyclopentol 1% to treat the inflammation and photophobia.

At follow-up one week later, our patient exhibited a robust response to topical corticosteroids. Both his photosensitivity and nummular keratitis had improved, although his visual acuity remained unchanged. Over the subsequent year, our patient had multiple, similar presentations to our office. His exam routinely consisted of photophobia, conjunctival injection and nummular keratitis which occurred in conjunction with arthritis and fever. His ocular inflammatory symptoms resolved quickly with topical prednisolone acetate and cyclopentol 1%.

Despite a satisfactory inflammatory response to topical corticosteroid, with each successive flair-up of disease our patient had progressive corneal scarring and worsening of his vision. Approximately 1 year after our initial encounter, the decision was made to begin treatment with simvastatin (10 mg/day). We saw him once while on this treatment regimen; he was still experiencing disease flairs, elevated laboratory values (ESR = 39 mm/h, CRP = 20.5 mg/L), and ocular complaints. He noted photophobia, intermittent pain and redness and decreased visual acuity.

Despite this regimen, he eventually developed a large central corneal scar in the right eye located in the region of recurrent keratopathy. Visual acuity in this eye consequently decreased to 20/80. At this point, his rheumatologists began infliximab infusions (7.8 mg/kg for a total of 600 mg) every 8 weeks. Due to concern that his severe arthritis may lead to permanent joint damage, weekly methotrexate (20 mg) was also a part of his regimen.

After eight cycles of therapy, his ocular exam was remarkably improved. He no longer complained of photophobia. On exam, both eyes had clear conjunctiva, the central corneal opacities had resolved, and despite residual corneal scarring, his uncorrected visual acuity had returned to 20/20 in both eyes [see Additional file 1, 2]. He no longer needed artificial tears and reported no ocular complaints since initiation of infliximab 12 months prior.

## Discussion

To date, our review of the literature revealed no reported cases of conjunctivitis or keratitis associated with HIDS. However, ocular surface manifestations of HIDS may be underreported. At the referral center that maintains the world's largest database of patients with HIDS based in Nijmegen, the Netherlands, many HIDS patients have mild conjunctivitis during attacks. These patients were not evaluated by ophthalmologists and therefore, it is possible that their complaints may reflect undiagnosed ocular inflammation attributable to HIDS (Dr. Anna Simon, personal communication).

Over the course of three years, our patient repeatedly presented with complaints of "red eyes" and photophobia. His ocular irritation was initially attributed to viral conjunctivitis. By the time the patient was seen in our clinic, he already demonstrated corneal scarring and secondary vision loss. Throughout his history, our patient continued to show signs of corneal inflammation despite treatment with ibuprofen, steroids and simvastatin. While his keratitis responded to topical corticosteroids, his vision never fully improved. It was not until initiation of infliximab therapy that his vision improved to 20/20. The complete resolution of his keratopathy with an anti-TNF-α agent suggests the drug works not only to reduce the frequency of HIDS flares, but also to decrease inflammatory mediators that cause damage occurring between attacks.

There currently is no standardized treatment regimen for HIDS. Traditional anti-inflammatory drugs (e.g. steroids) have moderate effects on tempering attacks. Given the role of mevalonate kinase in cholesterol biosynthesis, inhibition of HMG-CoA reductase with statins has been theorized to reduce the frequency of inflammatory attacks. One study did show simvastatin effectively lowered the duration of illness by reducing the frequency of febrile attacks, but its ability to prevent end-organ damage (arthritis, skin lesions, hepatospenomegaly, etc.) has been less satisfactory.[6] In fact, in our case we saw no improvement in our patient's ocular symptoms on simvastatin therapy. Interleukin-1 (IL-1) receptor antagonists (e.g. anakinra) have been used with reported success in vaccine-induced models of HIDS. They are thought to limit T-cell mediated immunity and cytokine production, especially during attacks. [7] Isoprene deficiency also leads to an excessive amount of proinflammatory cytokines and a downstream increase in plasma levels of TNF-α. Therefore, anti-TNF-α agents are being used in patients who continue to demonstrate end-organ damage despite treatment with other agents.[8] Results are mixed, but include a decrease in acute-phase response, improvement in clinical symptoms, and cessation of febrile attacks.[9,10]

Given the chronicity of the keratitis and associated corneal scarring with visual loss observed in our patient, we recommend that patients with HIDS and non-specific ocular complaints be evaluated and managed by an ophthalmologist. Furthermore, because of the recurrent nature of the keratopathy, good response to topical therapy, and the potential for visual loss, we recommend judicious use of topical corticosteroids to suppress the ocular surface inflammation in patients with HIDS. In cases such as ours, where vision loss was persistent despite good acute response to topical corticosteroids, we suggest consideration of systemic biologic therapy. Because the TNF-α antagonist specifically counteracts the increased acute-phase response seen in HIDS, it may be better suited than simvastatin or ibuprofen when end-organ damage, such as corneal scarring, is apparent.

## Competing interests

The authors declare that they have no competing interests.

## Authors' contributions

CLK: reviewed the chart, relevant literature and drafted the manuscript. SMC: examined the patient and reviewed drafts. All authors read and approved the final manuscript.

## Consent

Written informed consent was obtained from the patient's parents for publication of this case report. A copy of the written consent is available for review by the Editor-in-Chief of this journal.

## Supplementary Material

Additional file 1 **Close-up corneal photograph taken after resolution of keratopathy following treatment with infliximab**. Note residual stromal opacities at the 3 o'clock position. Click here for file

Additional file 2 **Zoomed out view of resolved keratopathy**. Unimpressive picture of a residual iron line, all that remains of what was a rather inflammed cornea. Click here for file
