# Supplementary figures and images for: Nummular keratopathy in a patient with Hyper-IgD Syndrome
Source: Pediatr Rheumatol Online J. 2009 Aug 5;7:14. doi: 10.1186/1546-0096-7-14 (PMC2731058; doi:10.1186/1546-0096-7-14)

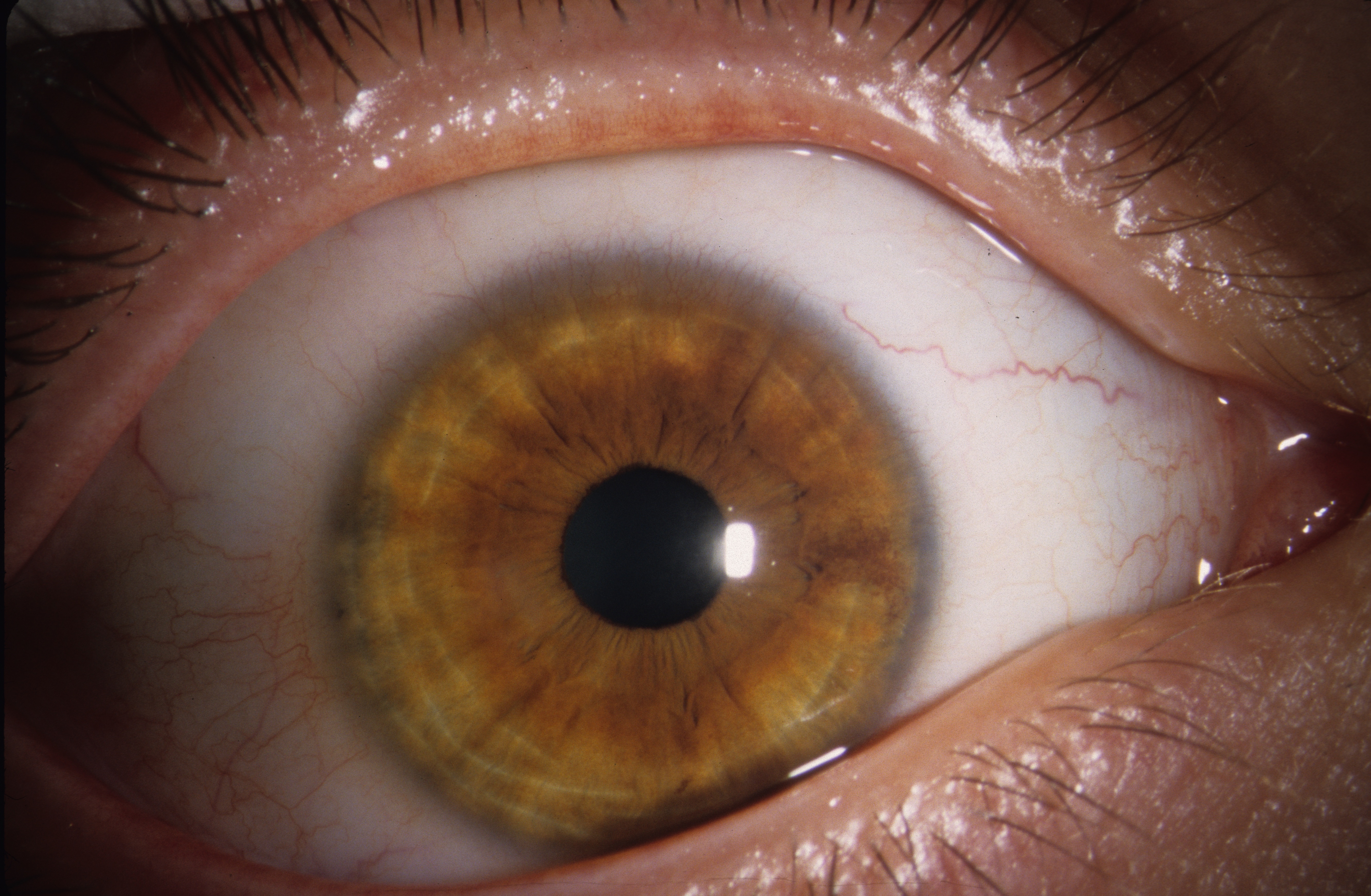

Supplement: Additional file 1 — Close-up corneal photograph taken after resolution of keratopathy following treatment with infliximab. Note residual stromal opacities at the 3 o'clock position. [file 1546-0096-7-14-S1.jpeg]

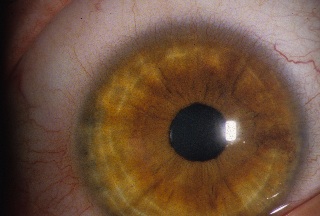

Supplement: Additional file 2 — Zoomed out view of resolved keratopathy. Unimpressive picture of a residual iron line, all that remains of what was a rather inflammed cornea. [file 1546-0096-7-14-S2.jpeg]
